# Behavioral changes in *FPR2/ALX* and *Chemr23* receptor knockout mice are exacerbated by prenatal alcohol exposure

**DOI:** 10.3389/fnins.2023.1187220

**Published:** 2023-07-06

**Authors:** Sandra M. Mooney, Elanaria Billings, Madison McNew, Carolyn A. Munson, Saame R. Shaikh, Susan M. Smith

**Affiliations:** ^1^Nutrition Research Institute, University of North Carolina at Chapel Hill, Kannapolis, NC, United States; ^2^Department of Nutrition, University of North Carolina at Chapel Hill, Kannapolis, NC, United States

**Keywords:** prenatal alcohol exposure, brain development, cognition, behavior, omega-3 polyunsaturated fatty acid, specialized pro-resolving mediator, Fetal Alcohol Spectrum Disorder, inflammation

## Abstract

**Introduction:**

Prenatal alcohol exposure (PAE) causes neuroinflammation that may contribute to the pathophysiology underlying Fetal Alcohol Spectrum Disorder. Supplementation with omega-3 polyunsaturated fatty acids (PUFAs) has shown success in mitigating effects of PAE in animal models, however, the underlying mechanisms are unknown. Some PUFA metabolites, specialized pro-resolving mediators (SPMs), play a role in the resolution phase of inflammation, and receptors for these are in the brain.

**Methods:**

To test the hypothesis that the SPM receptors FPR2 and ChemR23 play a role in PAE-induced behavioral deficits, we exposed pregnant wild-type (WT) and knockout (KO) mice to alcohol in late gestation and behaviorally tested male and female offspring as adolescents and young adults.

**Results:**

Maternal and fetal outcomes were not different among genotypes, however, growth and behavioral phenotypes in the offspring did differ and the effects of PAE were unique to each line. In the absence of PAE, ChemR23 KO animals showed decreased anxiety-like behavior on the elevated plus maze and FPR2 KO had poor grip strength and low activity compared to age-matched WT mice. WT mice showed improved performance on fear conditioning between adolescence and young adulthood, this was not seen in either KO.

**Discussion:**

This PAE model has subtle effects on WT behavior with lower activity levels in young adults, decreased grip strength in males between test ages, and decreased response to the fear cue indicating an effect of alcohol exposure on learning. The PAE-mediated decreased response to the fear cue was also seen in ChemR23 KO but not FPR2 KO mice, and PAE worsened performance of adolescent FPR2 KO mice on grip strength and activity. Collectively, these findings provide mechanistic insight into how PUFAs could act to attenuate cognitive impairments caused by PAE.

## 1. Introduction

At least 13.5% of pregnant women report using alcohol and ~ 5% report binge drinking (Gosdin et al., 2022). The incidence of Fetal Alcohol Spectrum Disorders (FASDs), a potential consequence of alcohol exposure, in the US is estimated at 2–5% (May et al., 2018). Prenatal alcohol exposure (PAE) causes behavioral effects in humans and in animal models including in learning and memory (Hoyme et al., 2016; Marquardt and Brigman, 2016); these outcomes persist through the lifespan but may present differently at different ages (e.g., Day and Richardson, 2004; Goldschmidt et al., 2019; Jacobson et al., 2021; Smith et al., 2022). At this time, little is known about mechanisms that underlie the risk and resilience factors affecting PAE, including nutrition.

Supplementation with omega-3 polyunsaturated fatty acids (PUFAs) has shown success in mitigating effects of PAE in animal models. We showed that postnatal supplementation with the omega-3 PUFA docosahexaenoic acid (DHA) improved prenatal alcohol-induced deficits in social behaviors in a rat model of FASD (Wellmann et al., 2015). Others have shown that maternal supplementation with lipids enriched with combinations of omega-3 PUFAs improve alcohol effects on sensory development, hyperactivity, and hippocampal synaptic plasticity (Wainwright et al., 1990; Patten et al., 2013; Balaszczuk et al., 2019). Interestingly, in a study in humans with FASDs we identified a SNP in the fatty acid desaturase gene *FADS2* that was associated with better performance on a memory test, supporting the idea that PUFAs may also modulate outcomes in humans following PAE (Smith et al., 2021). However, it is unknown how omega-3 PUFAs might confer such a benefit.

Candidate mechanisms include through their anti-inflammatory and/or pro-resolution activities. Alcohol is a potent pro-inflammatory agent, and PAE causes both systemic and neuroinflammation, and in animal models this inflammation can be persistent (Cantacorps et al., 2017; Komada et al., 2017; Bodnar et al., 2020; Gano et al., 2020). With respect to the brain, PAE elicits an activated microglial phenotype, astrogliosis, and pro-inflammatory cytokine production in the pre-weaning rat (Topper et al., 2015; Bodnar et al., 2016; García-Baos et al., 2021). PAE can also prime the brain for persistent neuroinflammation and hypersensitivity to microglial and astrocytic activation later in life (Cantacorps et al., 2017; Pascual et al., 2017; Chastain et al., 2019; García-Baos et al., 2021), and loss-of-function in the Toll-like receptor 4 (TLR4) that activates these inflammatory responses normalizes these behavioral deficits in the PAE offspring (Pascual et al., 2017). We recently reported that hippocampal microglial morphology indicative of greater microglial activation was still apparent at ~1 year of age in a mouse PAE model, and that this shows a trend to correlate with poorer performance on a recognition memory test (Walter et al., 2022).

Recent discoveries show that omega-3 PUFAs can also resolve inflammation (see review Dyall et al., 2022). Oxy-metabolites of long-chain PUFAs, especially those derived from eicosapentanoic acid (EPA) and DHA, act as short-lived, short-range lipid mediators that both initiate and resolve the intensity and duration of an inflammatory response (Serhan, 2014). Whereas prostaglandins, leukotrienes, and cytokines generally promote inflammation, the small molecules collectively known as specialized pro-resolving mediators (SPMs), serve to actively resolve that response. SPMs include resolvins, protectins, maresins, and lipoxins, all of which are derived from either omega-3 or omega-6 PUFAs. SPMs bind to specific G-protein coupled membrane receptors to suppress inflammatory signals and activate anti-inflammatory signaling cascades (Han et al., 2021). Of these, the formyl peptide receptor 2 (FPR2, aka FPR2/ALX) and the chemerin receptor (chemokine-like receptor 1 (CMKLR1) aka Chemerin1 aka ChemR23) play pivotal roles in mediating this “resolution pharmacology” (Mastromarino et al., 2020; Perretti and Godson, 2020; Qin et al., 2022). Ligands for both are derived from omega-3 PUFAs; ligands for FPR2 include the D-series resolvin 1, RvD1, derived from DHA (Krishnamoorthy et al., 2010) and for ChemR23 the E-series resolvin 1, RvE1 derived from EPA (Arita et al., 2005). Although their downstream signaling cascades are incompletely understood, resolvin binding to its receptor is shown to be both anti-inflammatory and pro-resolving of inflammation (Li et al., 2020; Anand et al., 2022).

Little is known about SPM actions in the brain. The Allen Brain Atlas shows that FPR2 and ChemR23 are expressed in multiple regions in adult mouse brain^1^,^2^ (see also Zhang et al., 2014) and that they are primarily found in microglia, consistent with these cells’ inflammatory role. They are also detected in neurons, oligodendrocytes, and astrocytes, albeit at much lower levels. In adult rat brain FPR2 has differential expression across brain regions and is highly expressed in regions important for cognitive behaviors, including prefrontal cortex and hippocampus (Ho et al., 2018) but differential localization of ChemR23 in the rodent brain has not been reported. Relatively little is known about the potential role of these receptors in behavior. In one study FPR2 KO animals show increased activity, increased time in the light side of the light–dark box, and increased recognition memory (using the novel object recognition test) than WT, interpreted as evidence of lower anxiety (Gallo et al., 2014).

To test the hypothesis that the SPM receptors FPR2 and ChemR23 play a role in PAE-induced behavioral deficits, we exposed pregnant wild-type and knockout mice to alcohol for 5 days in late gestation and tested male and female offspring on a variety of behavior tests as adolescents and young adults.

## 2. Methods

### 2.1. Animals

Wild-type (WT) C57BL/6J from Jackson Labs (Bar Harbor, ME) and FPR2 KO and ChemR23 KO mouse strains were used. KO strains were generated by the UNC Animal Models Core from WT (C57BL/6J) mice using CRISPR/Cas9-mediated genome editing. Exon 3 was deleted from ChemR23 KO mice and exon 2 from FPR2 KOs. The guide RNAs used in the genome editing for ChemR23 KO mice were 5sg81T (protospacer sequence 5′-GAGATCGTTCACAACCC-3′) and 3sg81T (protospacer sequence 5′-gCGGCCCAGGGACGCCTA-3′) and for FPR2 KO were 5sg74B (protospacer sequence 5′-gATACCACCTGCTACTAC-3′) and 3sg47T (protospacer sequence 5′-GAGCAATGCATATTCTC-3′). All other methods (cloning, transformation, progeny screening) were the same as described (Pal et al., 2020). Deletion of the relevant exon for each strain was verified by PCR using the primers described in Supplementary Figure S1. Mice were bred as WT-WT or KO-KO crosses to avoid potential competition between neonates of differing genotypes and to assure sufficient offspring of both sexes for the behavioral testing.

Dams consumed a fixed-nutrient diet (AIN-93G, TD. 94,045, Teklad Envigo, Indianapolis, IN; Reeves et al., 1993) from 3 weeks prior to mating through lactation. This diet contains 0.48% by weight of the omega-3 precursor alpha-linolenic acid but no EPA or DHA. Offspring consumed the same diet after weaning. Animals were kept in a temperature- and humidity-controlled environment with a 12:12 light-dak cycle (lights on at 7 a.m.). All procedures were approved by the David H. Murdock Research Institute.

Female mice were mated to genotype-matched males overnight with plug day designated embryonic day (E)0.5. On E13.5 through E17.5 dams received two daily oral gavages of 2.25 g/kg body weight alcohol given 2 h apart (total daily exposure 4.5 g/kg; alcohol-exposed, ALC) or iso-caloric maltodextrin (control, CON). After weaning on postnatal day (P)28, mice were housed with 2–3 same-sex littermates where possible.

Dam body weight was recorded at E0.5, E13.5, and E17.5; weight gain over the whole pregnancy and during the gavage period was calculated. Offspring were weighed and underwent behavior testing in adolescence (6 weeks of age; 6 wk) and again at young adulthood (14 wk). Mice were euthanized at ~16 wk by cardiac puncture under isoflurane anesthesia; body, brain, and liver weights were recorded. Final sample sizes are 8–16 animals from 5 to 10 litters and are shown in Table 1.

**Table 1:** Number of animals that underwent behavior testing (number of litters).

Genotype	Male CON	Male ALC	Female CON	Female ALC
WT	13 (7)	9 (5)	12 (7)	10 (5)
FPR2 KO	9 (5)	11 (6)	8 (5)	12 (6)
ChemR23 KO	16 (10)	11 (8)	12 (9)	11 (8)

ALC, alcohol-exposed; CON, control; FPR2, formyl peptide receptor 2; KO, knockout; WT, wild type.

### 2.2. Blood alcohol concentrations

BACs were determined in FPR2 KO and ChemR23 KO non-pregnant females. Samples were analyzed using the Analox GM7 Micro-Stat (Analox Technologies, Atlanta, GA) and compared with published WT data (Kwan et al., 2020).

### 2.3. Behavior testing

Testing was typically performed in the order described. A maximum of two animals of each sex were used from a given litter, our previous work shows that litter contributes significant variability to body weight (~25%) but not to behavior outcomes (typically <10%) (Smith et al., 2022). Animals were tested at both 6 and 14 wk.

#### 2.3.1. Elevated plus maze

The elevated plus maze (EPM) has 4 arms (35 cm in length and 5 cm in width) and a center area (5 cm × 5 cm). Two arms have walls that are 20 cm high and two arms are open. The maze stand is 61 cm high. The mouse is placed in the center of the maze facing an open arm and the center-point is tracked for 5 min using Ethovision XT software (Noldus, Leesburg, VA). The software records time spent in each of the arms and in the center. Distance traveled in the maze, percent time in closed arms, and number of entries into open arms are reported.

#### 2.3.2. Grip strength

The assessment is done using a grip strength meter (Harvard Apparatus, Holliston, MA) that has a grid that is connected to a sensor. The animal is allowed to grab the grid with its forelimbs and is then pulled gently backwards. The force reading right before the animal loses grip is recorded as highest tension (grams). Three successive trials are measured and the highest tension recorded is normalized to the animal’s body weight.

#### 2.3.3. Accelerating rotarod

Mice were placed on a rotarod (BioSeb, Pinellas Park, FL) which accelerates from 3 revolutions per minute (rpm) to 30 rpm over 5 min (300 s). Mice were given an initial training trial and after a 45 s intertrial interval, the 5 min test trial. Latency (s) to fall from the rotating barrel was recorded.

#### 2.3.4. Novel object recognition

Mice were placed facing the wall in a rectangular arena (40 cm × 40 cm × 29.5 cm) and allowed to explore for 5 min. Total distance traveled during this time was determined using Ethovision software and is reported as activity. For the NOR test, two of the same object were introduced in the arena and the mice were left to explore for 3 min. Twenty four hour later, mice were placed in the arena where one of the familiar objects was substituted with a novel object, and the mouse was left to explore the objects for 3 min. Time exploring objects was determined by Ethovision software when the mouse’s nose was within 2 cm of the object. Percent time exploring the novel object was calculated as time spent with novel/total time with objects.

#### 2.3.5. Auditory cued fear conditioning

This is a 3-day procedure performed as previously described (Video Fear Sound Attenuating Cubicles, Med Associates, Fairfax, VT) (Mooney et al., 2021; Smith et al., 2022; Walter et al., 2022). Briefly, on Day 1 animals acquire fear conditioning through 4 exposures to a 30 s tone that co-terminates with a 2 s foot shock. Day 2 is a test of contextual conditioning in which animals are in the same chamber as Day 1 for 5 min. Day 3 tests cued conditioning; animals are in a chamber with different features for 5 min but hear the tone (cue) used on Day 1 at beginning at 2 min. For all outcomes, percent time freezing is determined by Ethovision software.

### 2.4. Statistical analysis

Group means and standard deviations were generated for each dependent measure. Between-group differences in maternal and litter outcomes and post-mortem tissue weights were tested using two-way analysis of variance (ANOVA; genotype and group). Because we know that body and organ weights differ by sex, two-way ANOVAs (genotype, exposure) were used for analysis of these data. To directly test for sex differences in behavior, three-way repeated measures (RM) ANOVAs (genotype, exposure, sex) were applied to those data. Alpha was set at <0.05, Bonferroni post-hoc tests were performed where significance was found, and trends are reported where *p* < 0.1. Associations between behavior and body weight and between grip strength and other behaviors were assessed using Pearson correlation analysis of the whole dataset and separately for each genotype to determine if associations were ubiquitous or unique. Analyses were done using SigmaPlot (Systat Software Inc., version 14.0, Palo Alto, CA) and SPSS (IBM SPSS Statistics, version 28, Armonk, NY).

## 3. Results

### 3.1. Dam and litter outcomes

Two-way ANOVA showed there was no difference among genotypes in dam weight gain through pregnancy, in the mean number of pups per litter at weaning, or the percent that were male (largest *F* = 1.895, smallest *p* = 0.177; Table 2). Weight gain during the gavage period showed a trend to be lower for ALC dams (*F*_1,35_ = 3.865, *p* = 0.057) across the genotypes. Dam body weights were different among genotypes (*F*_2,35_ = 9.385, *p* < 0.001) and exposure groups at E0.5 (*F*_1,35_ = 4.473, *p* = 0.042); ChemR23 KO dams weighed more than WT (*p* = 0.002) or FPR2 KO (*p* = 0.003), likely because they were older at initiation of breeding, and dams assigned to CON were heavier than those assigned to ALC. There was an effect of genotype at E13.5 (*F*_2,35_ = 3.705, *p* = 0.035); ChemR23 KO dams showed a trend to weigh more than WT (*p* = 0.073) and FPR2 KO (*p* = 0.070) mice. No differences were seen at E17.5.

**Table 2:** Dam and litter outcomes.

Group (n)	Dam weight E0.5 (g)	Dam weight E13.5 (g)	Dam weight E17.5 (g)	Pregnancy weight gain (g)	Gavage weight gain (g)	Number of pups	% male
WT CON (7)	19.67 ± 1.16^a^	26.48 ± 1.57^a^	32.50 ± 2.06	12.84 ± 2.36	5.86 ± 2.04	5.9 ± 2.0	52.2 ± 16.6
WT ALC (5)	19.05 ± 1.24^a^	26.72 ± 1.17^a^	30.79 ± 1.91	11.75 ± 1.45	4.07 ± 1.57	5.0 ± 1.2	44.0 ± 18.8
FPR2 CON (5)	19.47 ± 0.66^a^	26.41 ± 1.41^a^	32.30 ± 2.16	12.83 ± 2.12	5.89 ± 1.16	5.2 ± 1.3	53.8 ± 21.1
FPR2 ALC (6)	19.36 ± 0.76^a^	26.96 ± 1.39^a^	32.40 ± 2.25	13.04 ± 1.58	5.47 ± 0.91	5.5 ± 1.1	47.7 ± 17.8
ChemR23 CON (10)	**21.77 ± 1.41** ^**b**^	**28.47 ± 1.04** ^**b**^	33.35 ± 1.33	11.58 ± 2.32	4.88 ± 1.33	5.7 ± 2.5	59.0 ± 22.2
ChemR23 ALC (8)	**20.16 ± 1.17** ^**b**^	**27.37 ± 2.10** ^**b**^	32.17 ± 2.82	12.01 ± 2.41	4.79 ± 1.41	4.7 ± 1.0	45.8 ± 24.0

ALC, alcohol-exposed; CON, control; FPR2, formyl peptide receptor 2; KO, knockout; WT, wild type. Means that do not share a common superscript letter differ at *p* < 0.05. Significant *p*-values are bold.

### 3.2. Blood alcohol concentration

BAC in WT animals 30 min after the second gavage was 217 ± 21 mg/dL (Kwan et al., 2020), in FPR2 KO mice this was 261 ± 50 mg/dL and in ChemR23 KO was 243 ± 15 mg/dL. One way ANOVA showed no significant differences among genotypes (*F*_2,6_ = 1.384, *p* = 0.320). Comparison of BACs in pregnant and non-pregnant FPR2 KO mice showed no differences (Mooney, unpublished data).

### 3.3. Body weights of experimental animals

In males, the two-way RM ANOVA identified a significant interaction for age × genotype (*F*_2,63_ = 6.861, *p* = 0.002), as well as main effect of age (*F*_1,63_ = 1995.348, *p* < 0.001; Table 3). Male ChemR23 KO mice weighed less than WT at 6 wk (*p* < 0.001) and 14 wk (*p* = 0.005) and weighed less than FPR2 KO males at 6 wk (*p* < 0.001). As expected, all animals gained weight between 6 and 14 wk (*F*_2,63_ = 6.861, *p* = 0.002), but FPR2 KO mice gained significantly less weight during that period than did WT (*p* = 0.008) and ChemR23 KO (*p* = 0.004).

**Table 3:** Offspring outcomes.

Males	6 wk body weight (g)	14 wk body weight (g)	Weight gain (g)	16 wk body weight (g)	Liver weight (g)	Brain weight (g)*	Ratio brain weight to body weight
WT CON	20.26 ± 1.18^a^	27.08 ± 0.98^a^	6.82 ± 0.84^a^	27.73 ± 1.33	1.16 ± 0.18	0.42 ± 0.02	0.015 ± 0.001
WT ALC	21.15 ± 2.15^a^	27.81 ± 2.31^a^	6.66 ± 0.81^a^	27.29 ± 1.57	1.23 ± 0.08	0.44 ± 0.02	0.016 ± 0.001
FPR2 CON	21.12 ± 0.98^a^	26.48 ± 1.96^a^	**5.36 ± 1.59** ^**b**^	27.11 ± 2.06	1.17 ± 0.13	0.42 ± 0.01	0.016 ± 0.001
FPR2 ALC	21.19 ± 0.58^a^	27.05 ± 1.23^a^	**5.86 ± 1.02** ^**b**^	27.45 ± 1.39	1.13 ± 0.09	0.42 ± 0.02	0.015 ± 0.001
ChemR23 CON	**19.08 ± 0.96** ^**b**^	**25.94 ± 1.14** ^**b**^	6.86 ± 1.39^a^	26.51 ± 1.33	1.14 ± 0.09	0.41 ± 0.01	0.016 ± 0.001
ChemR23 ALC	**19.18 ± 2.15** ^**b**^	**25.88 ± 2.01** ^**b**^	6.70 ± 1.09^a^	26.95 ± 1.95	1.22 ± 0.09	0.43 ± 0.01	0.016 ± 0.001
Females	6 wk body weight (g)	14 wk body weight (g)	Weight gain (g)	16 wk body weight (g)	Liver weight (g)	Brain weight (g)	Ratio brain weight to body weight
WT CON	16.57 ± 0.87^a^	**21.17 ± 0.87** ^**a**^	**4.60 ± 0.69** ^**a**^	21.08 ± 0.98	0.83 ± 0.13^a^	0.45 ± 0.01^a^	0.021 ± 0.001
WT ALC	16.50 ± 1.03^a^	**20.45 ± 1.53** ^**a**^	**3.95 ± 1.06** ^**a**^	20.17 ± 1.80	0.85 ± 0.11^a^	0.45 ± 0.02^a^	0.022 ± 0.002
FPR2 CON	17.22 ± 0.68^a^	19.47 ± 0.69^b^	2.25 ± 0.46^b^	20.12 ± 0.91	0.77 ± 0.11^a^	0.44 ± 0.02^a^	0.022 ± 0.002
FPR2 ALC	16.93 ± 0.83^a^	19.16 ± 0.70^b^	2.23 ± 0.40^b^	19.61 ± 0.79	0.78 ± 0.03^a^	0.44 ± 0.02^a^	0.022 ± 0.001
ChemR23 CON	**16.12 ± 0.78** ^**b**^	19.96 ± 0.88^b^	**3.84 ± 0.74** ^**c**^	20.19 ± 1.03	**0.90 ± 0.08** ^**b**^	**0.42 ± 0.03** ^**b**^	0.021 ± 0.002
ChemR23 ALC	**16.17 ± 1.22** ^**b**^	19.54 ± 1.29^b^	**3.38 ± 0.47** ^**c**^	19.98 ± 1.54	0.78 ± 0.13^a^	**0.42 ± 0.02** ^**b**^	0.021 ± 0.002

ALC, alcohol-exposed; CON, control; FPR2, formyl peptide receptor 2; KO, knockout; WT, wild type. Means that do not share a common superscript letter differ at *p* < 0.05. Significant *p*-values are bold. See text for more information.

*Significant difference between exposure groups.

For females, there were significant interactions for age × exposure (*F*_1,59_ = 5.022, *p* = 0.029), age × genotype (*F*_2,59_ = 47.770, *p* < 0.001), and a main effect of age (*F*_1,59_ = 1585.674, *p* < 0.001; Table 3). At 14 wk there was a trend for ALC animals to weigh less than CON (*p* = 0.067). ChemR23 KO females weighed less than FPR2 KO at 6 wk (*p* = 0.005) but neither were different to WT, and at 14 wk FPR2 KO or ChemR23 KO females weighed less than WT (*p* < 0.001 and *p* = 0.004, respectively). As expected, all animals gained weight between 6 and 14 wk (*p* < 0.001). There were significant effects of genotype (*F*_2,59_ = 47.803, *p* < 0.001) and exposure (*F*_1,59_ = 5.020, *p* = 0.029) on weight gain; KO mice animals gained less weight than WT (FPR2 *p* < 0.001, ChemR23 *p* = 0.005), ChemR23 KO mice gained more weight than FPR2 KO (*p* < 0.001), and ALC animals gained less weight than CON (*p* = 0.029).

### 3.4. Tissue weights of experimental animals

Tissues were collected at ~16 wk (Table 3). In males, the brains of ALC animals weighed more than CON (*F*_1,63_ = 9.323, *p* = 0.003). Body weight at the time of collection was not different among the groups, neither was the brain weight to body weight ratio, or the liver weight. In females, brain weight was significantly different among the genotypes (*F*_2,58_ = 8.155, *p* < 0.001); it was lower in ChemR23 KO mice than either FPR2 KO (*p* = 0.042) or WT (*p* < 0.001). Body weight at collection showed a trend to be lower in ALC animals than CON (*p* = 0.082), but there were no effects on the brain weight to body weight ratio. Liver weight showed a genotype × exposure interaction (*F*_2,58_ = 3.249, *p* = 0.046); in ChemR23 KO mice, ALC animals had lower liver weight than CON (*p* = 0.006).

### 3.5. Behavior testing

#### 3.5.1. Elevated plus maze

**Figure 1:**
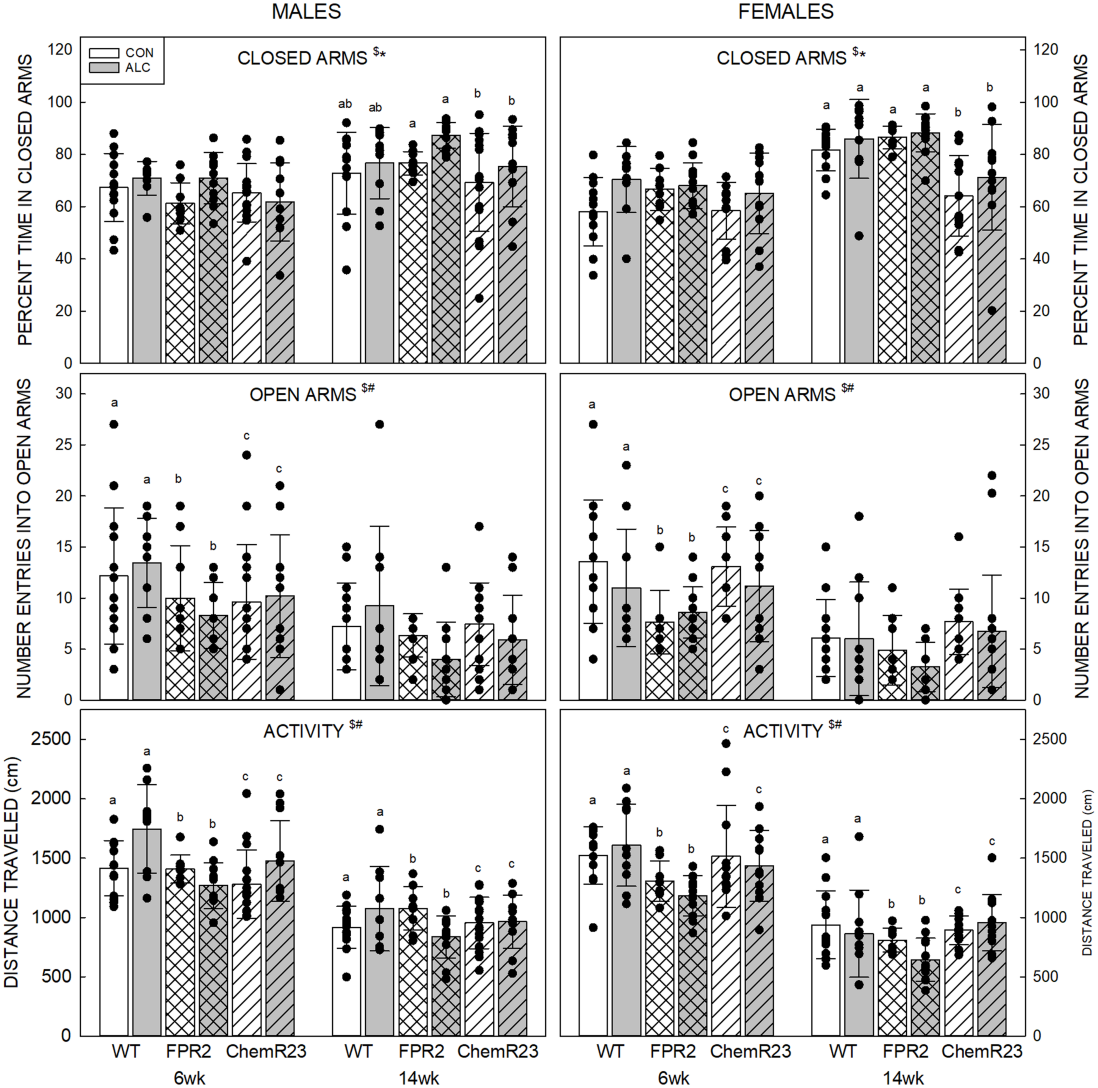
Elevated plus maze. (Top) Older animals spent more time in the closed arms than younger animals and ALC animals spent more time in the closed arms than CON. At 14 wk, ChemR23 females spent less time in closed arms than WT or FPR2 KO. (Middle) The number of entries into open arms was lower at 14 wk than 6 wk and was lower in FPR2 KO mice compared to WT or ChemR23 KO. (Bottom) Activity in the EPM (distance traveled during the test) decreased with age for all groups. Differences between genotypes were only seen in ALC mice where FPR2 KO-ALC mice moved less than WT or ChemR23 ALC mice. Data shown are mean and standard deviation. Comparisons across age are only made within genotype. Dots represent individual animals with *N* = 8–16 mice per group. Experimental group means that do not share a common superscript letter differ at *p* < 0.05; omission of superscript indicates no difference by genotype or exposure at that age. *Significant difference between exposure groups. ^#^Significant difference among genotypes. ^$^Significant difference between ages. ALC, alcohol-exposed; CON, control; EPM, elevated plus maze; FPR2, formyl peptide receptor 2; KO, knockout; WT, wild type.

Percent time in closed arms: Three-way RM ANOVA identified significant interactions for age × genotype × sex (*F*_2,122_ = 4.908, *p* = 0.009), age × genotype (*F*_2,122_ = 7.363, *p* < 0.001), age × sex (*F*_1,122_ = 5.034, *p* = 0.027), and significant effects of exposure (*F*_1,122_ = 7.771, *p* = 0.006) and age (*F*_1,122_ = 124.553, p < 0.001) (Figure 1). Overall, percent time in closed arms was higher in ALC mice than CON (*p* = 0.006) and was higher at 14 wk than 6 wk (*p* < 0.001). All genotypes and both sexes showed the decrease at 14 wk (all *p*s <0.001). No genotype differences were seen at 6 wk, but at 14 wk female ChemR23 KO mice spent less time in the closed arms than WT (*p* = 0.004) or FPR2 KO (*p* < 0.001) females.

Number of entries into open arms: The three-way RM ANOVA identified a significant effect of age (*F*_1,122_ = 109.591, *p* < 0.001) and genotype (*F*_2,122_ = 6.922, *p* = 0.001). The number of entries into open arms decreased between 6 and 14 wk (*p* < 0.001) and was lower in FPR2 KO compared with WT (*p* = 0.001) and ChemR23 KO (*p* = 0.023) but was not different between WT and ChemR23 KO mice.

Activity in EPM: Three-way RM ANOVA identified significant interactions for age × genotype (*F*_2,122_ = 4.588, *p* = 0.012), age × sex (*F*_1,122_ = 5.473, *p* = 0.012), exposure × genotype (*F*_2,122_ = 5.012, *p* = 0.008), a trend for age × exposure (*F*_1,122_ = 3.024, *p* = 0.085), and significant effects of genotype (*F*_2,122_ = 8.338, *p* < 0.001) and age (*F*_1,122_ = 441.426, *p* < 0.001). Animals from all genotypes and both sexes traveled further at 6 wk than 14 wk (all *p*s <0.001). In the CON group, there were no genotype differences, but in the ALC group FPR2 KO mice traveled less than WT (*p* < 0.001) and ChemR23 KO (*p* = 0.002).

#### 3.5.2. Grip strength

**Figure 2:**
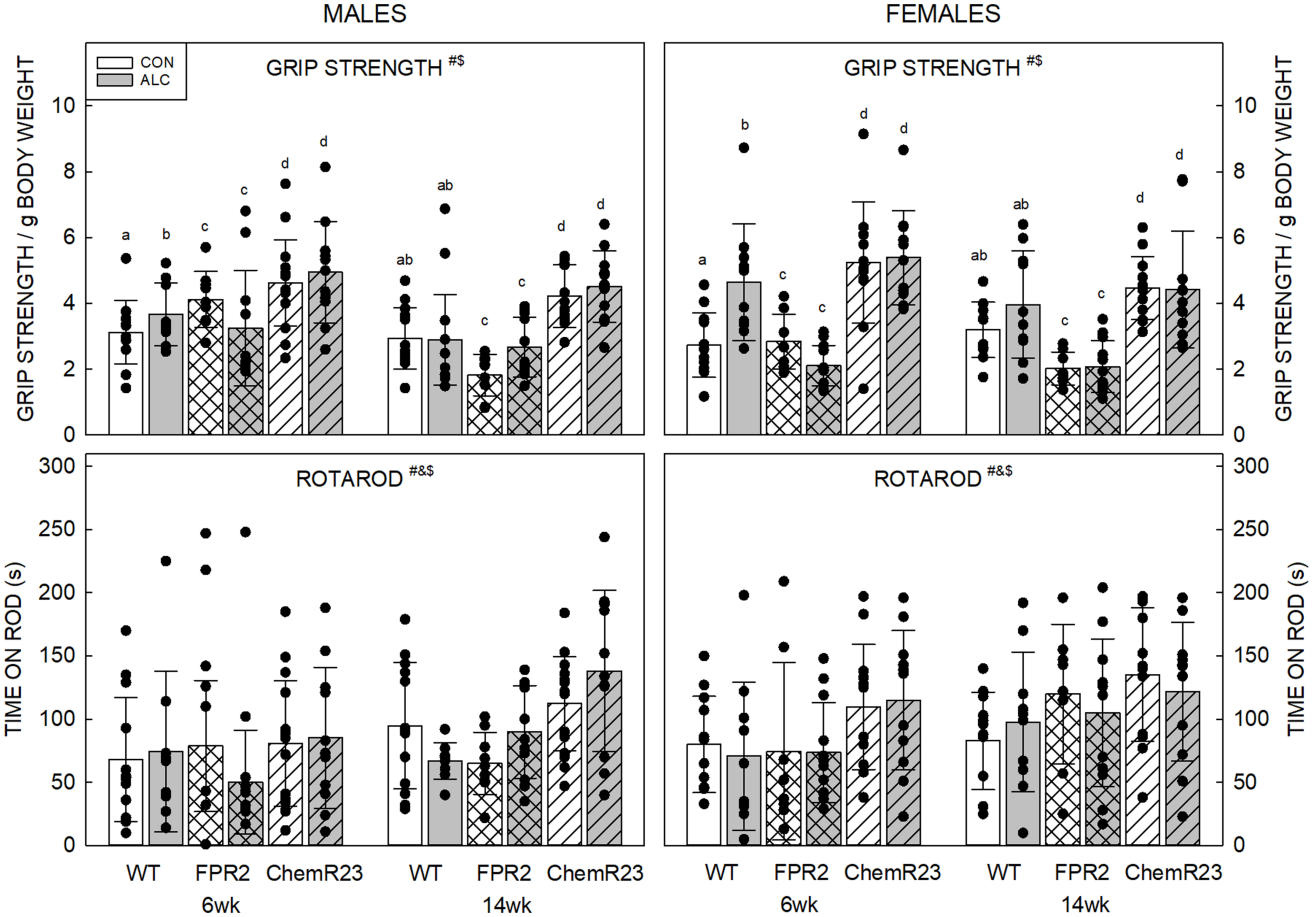
Motor outcomes. (Top) There were significant effects of age (*F*_1,122_ = 22.902, *p* < 0.001) and genotype (*F*_2,122_ = 56.184, *p* < 0.001), and interactions of age × genotype × exposure (*F*_2,122_ = 5.304, *p* = 0.006) and genotype × sex (*F*_2,122_ = 4.607, *p* = 0.012). Grip strength decreased with age and differed among the genotypes. Overall, grip strength was highest in ChemR23 KO and was not affected by ALC in these KOs. In WT mice, grip strength was higher in ALC mice than CON at 6 wk. FPR2 KO mice had the lowest grip strength and showed a trend for this to be lower in ALC mice than CON at 6 wk. (Bottom) There were significant effects of age (*F*_1,122_ = 16.591, *p* < 0.001), genotype (*F*_2,122_ = 10.417, *p* < 0.001), and sex (*F*_1,122_ = 5.045, *p* = 0.026). Females spent more time on the rod than males, and ChemR23 KO mice did better than WT or FPR2 KO. Both KO lines spent more time on the rod at 14 wk than at 6 wk, this was not seen in WT mice. Data shown are mean and standard deviation. Comparisons across age are only made within genotype. Dots represent individual animals with *N* = 8–16 mice per group. Experimental group means that do not share a common superscript letter differ at *p* < 0.05; omission of superscript indicates no difference by genotype or exposure at that age. *Significant difference between exposure groups. ^#^Significant difference among genotypes. ^&^Significant difference between sexes. ^$^Significant difference between ages. ALC, alcohol-exposed; CON, control; FPR2, formyl peptide receptor 2; KO, knockout; WT, wild type.

Three-way RM ANOVA identified significant interactions for age × genotype × exposure (*F*_2,122_ = 5.304, *p* = 0.006) and genotype × sex (*F*_2,122_ = 4.607, *p* = 0.012), trends for interaction of age × genotype × sex (*F*_2,122_ = 2.734, *p* = 0.069) and genotype × exposure (*F*_2,122_ = 2.752, *p* = 0.068), and significant effects of age (*F*_1,122_ = 22.902, *p* < 0.001) and genotype (*F*_2,122_ = 56.184, *p* < 0.001) (Figure 2).

For all groups, mice did better on grip strength at 6 wk than 14 wk (*p* < 0.001), possibly reflecting a higher body weight at the latter age. All genotypes were different to each other; ChemR23 KO animals did better than WT, and both did better than FPR2 KO animals (all *p*s <0.001).

ALC interacted with genotype and age; for WT ALC animals, grip strength was higher than CON at 6 wk (*p* = 0.003) but did not differ from WT CON at 14 wk. FPR2 KO mice showed a trend for effect of exposure at 6 wk (*p* = 0.059) where ALC mice did worse than CON; this was not seen at 14 wk. ChemR23 KO mice did not show effects of exposure at either age.

Investigation of the genotype × sex effect showed that FPR2 KO males did better than FPR2 KO females (*p* = 0.022) and WT females showed a trend to do better than WT males (*p* = 0.098). ChemR23 KO mice did not show sex differences at either age.

#### 3.5.3. Rotarod

Three-way RM ANOVA identified a trend for an interaction between age × genotype × sex (*F*_2,122_ = 2.927, *p* = 0.057) and effects of age (*F*_1,122_ = 16.591, *p* < 0.001), genotype (*F*_2,122_ = 10.417, *p* < 0.001), and sex (*F*_1,122_ = 5.045, *p* = 0.026) (Figure 2). Mice spent more time on the rotarod at 14 wk than 6 wk (*p* < 0.001), females outperformed males (*p* = 0.026), and ChemR23 KO mice did better than WT (*p* < 0.001) or FPR2 KO (*p* = 0.001). The time WT animals spent on the rotarod did not change with age, but FPR2 females (*p* = 0.008) and ChemR23 males (*p* < 0.001) significantly improved their performance between 6 and 14 wk.

#### 3.5.4. Novel object recognition

**Figure 3:**
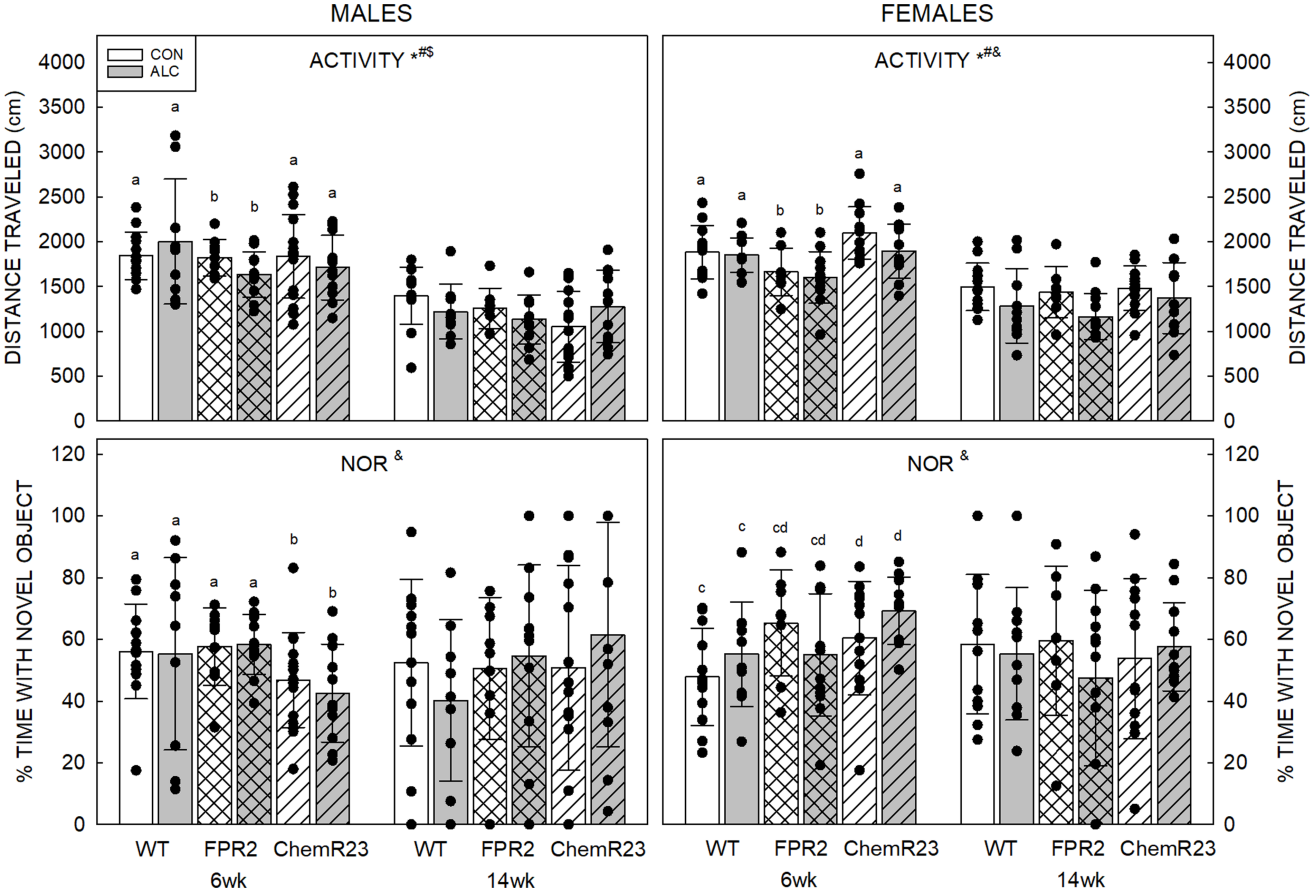
Activity and novel object recognition. (Top) There were significant effects of age (*F*_1,122_ = 213.628, *p* < 0.001), genotype (*F*_2,122_ = 3.930, *p* = 0.022), and exposure (*F*_1,122_ = 3.929, *p* = 0.050), and an interaction between age × genotype × exposure (*F*_2,122_ = 4.056, *p* = 0.020). Distance traveled in an empty arena was less in ALC animals than CON, less at 14 wk than 6 wk, and less in FPR2 KO mice than WT. Activity at 14 wk was not affected by genotype or exposure. (Bottom) There was a significant interaction between age × genotype × sex (*F*_2,122_ = 3.460, *p* = 0.035). At 6 wk, ChemR23 KO males spent less time with the novel object than FPR2 KO and showed a trend (*p* = 0.087) to spend less time than WT males, whereas 6 wk ChemR23 KO females spent more time with the novel object than WT females. There was an overall trend for females to spend more time with the novel object than did males. Data shown are mean and standard deviation. Comparisons across age are only made within genotype. Dots represent individual animals with *N* = 8–16 mice per group. Experimental group means that do not share a common superscript letter differ at *p* < 0.05; omission of superscript indicates no difference by genotype or exposure at that age. *Significant difference between exposure groups. ^#^Significant difference among genotypes. ^&^Significant difference between sexes. ^$^Significant difference between ages. ALC, alcohol-exposed; CON, control; FPR2, formyl peptide receptor 2; KO, knockout; WT, wild type.

For activity in the NOR arena, three-way RM ANOVA identified a significant interaction between age × genotype × exposure (*F*_2,122_ = 4.056, *p* = 0.020), trends for genotype × sex (*F*_2,122_ = 2.839, *p* = 0.062) and age × sex (*F*_1,122_ = 3.067, *p* = 0.082) interactions, significant effects of age (*F*_1,122_ = 213.628, *p* < 0.001), genotype (*F*_2,122_ = 3.930, *p* = 0.022), and exposure (*F*_1,122_ = 3.929, *p* = 0.050), and a trend for an effect of sex (*F*_1,122_ = 2.839, *p* = 0.069) (Figure 3).

Distance traveled was less in ALC mice than CON (*p* = 0.050), less at 14 wk than 6 wk for all groups (*p* < 0.001) and showed a trend to be less in males than females (*p* = 0.069). FPR2 KO animals were significantly less active than WT (*p* = 0.027) and showed a trend to be less active than ChemR23 KO mice (*p* = 0.092). Sex differences were apparent at 14 wk (*p* = 0.010), where females traveled further than males.

CON animals did not show effects of age or exposure, but ALC animals did. At 6 wk, ALC FPR2 KO mice were less active than ALC WT (*p* = 0.016), and both ALC WT (*p* = 0.052) and ALC FPR2 KO (*p* = 0.059) showed trends for less activity at 14 wk than 6 wk.

For percent time with the novel object, three-way RM ANOVA identified a significant interaction between age × genotype × sex (*F*_2,122_ = 3.460, *p* = 0.035). In 6 wk males, ChemR23 KO mice spent significantly less time with the novel object than FPR2 KO (*p* = 0.030) and show a trend to less compared with WT (*p* = 0.087). In 6 wk females, ChemR23 KO mice spend more time with the novel object than WT animals (*p* = 0.031). No differences among groups were seen at 14 wk but there was an overall weak trend for females to spend more time with the novel object than males (*F*_1,122_ = 3.049, *p* = 0.083).

#### 3.5.5. Auditory cued fear conditioning

**Figure 4:**
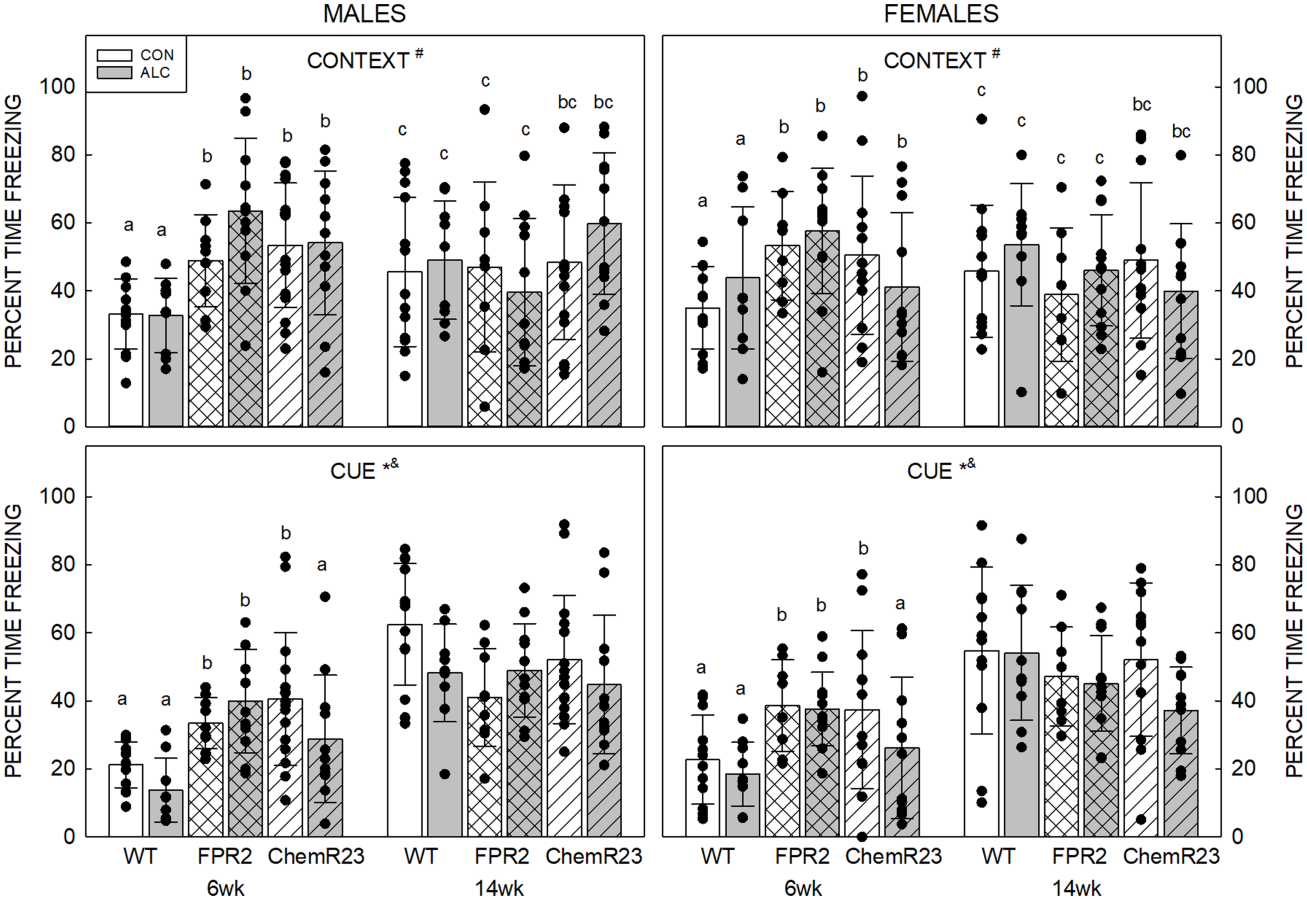
Auditory cued fear conditioning. (Top) Context. There is an age × genotype interaction (*F*_2,122_ = 20.761, *p* < 0.001), such that at 6 wk, FPR2 and ChemR23 KO mice show more freezing to the context compared to WT. WT mice show an age-related increase in freezing at 14 wk; however, FPR2 KO mice show a decrease in freezing with age, and there is no age-related change in ChemR23 KO mice. (Bottom) In response to the cue, there were significant effects of age (*F*_1,122_ = 116.489, *p* < 0.001) and exposure (*F*_1,122_ = 4.659, *p* = 0.033), and interactions between age × genotype (*F*_2,122_ = 22.566, *p* < 0.001) and genotype × exposure (*F*_2,122_ = 3.110, *p* = 0.048). ALC animals freeze less than CON and younger animals freeze less than older. KO mice freeze more than WT at 6 wk and this shows a trend to be lower in KO than WT at 14 wk. ALC ChemR23 KO mice freeze less than their CON. There was no significant effect of genotype or exposure at 14 wk. Data shown are mean and standard deviation. Comparisons across age are only made within genotype. Dots represent individual animals with *N* = 8–16 mice per group. Experimental group means that do not share a common superscript letter differ at *p* < 0.05; omission of superscript indicates no difference by genotype or exposure at that age. *Significant difference between exposure groups. ^#^Significant difference among genotypes. ^&^Significant difference between sexes. ^$^Significant difference between ages. ALC, alcohol-exposed; CON, control; FPR2, formyl peptide receptor 2; KO, knockout; WT, wild type.

Context: Three-way RM ANOVA identified a significant age × genotype interaction (*F*_2,122_ = 20.761, *p* < 0.001) as well as trends for an interaction between age × genotype × exposure × sex (*F*_2,122_ = 2.954, *p* = 0.056) and effect of genotype (*F*_2,122_ = 2.461, *p* = 0.090) (Figure 4).

At 6 wk FPR2 KO (*p* < 0.001) and ChemR23 KO (*p* = 0.001) mice freeze more than WT, and the KO strains did not different to each other. Both WT and FPR2 KO animals show changes in freezing with age; this decreased in FPR2 KO (*p* < 0.001) but increased in WT (*p* < 0.001) at 14 wk compared with 6 wk.

At 6 wk CON ChemR23 KO males freeze more than WT (*p* = 0.010), and CON FPR2 KO females show a trend to freeze more than WT (*p* = 0.083), but there were no differences at 14 wk. In ALC animals, 6 wk FPR2 KO (*p* < 0.001) or ChemR23 KO (*p* = 0.028) males freeze more than WT, and at 14 wk, there is a trend for more freezing in ChemR23 KO males than FPR2 KO (*p* = 0.073). At 6 wk ALC ChemR23 KO females show a trend for less freezing than FPR2 KO (*p* = 0.091) and there are no differences among female groups at 14 wk.

Cue: Three-way RM ANOVA identified significant interactions for age × genotype (*F*_2,122_ = 22.566, *p* < 0.001) and genotype × exposure (*F*_2,122_ = 3.110, *p* = 0.048), and effects of age (*F*_1,122_ = 116.489, *p* < 0.001) and exposure (*F*_1,122_ = 4.659, *p* = 0.033) (Figure 4). Overall, younger animals freeze less than older (*p* < 0.001) and ALC mice freeze less than CON mice (*p* = 0.033). At 6 wk, KO mice freeze more than WT mice (both *p*s <0.001), and the KO were not different to each other, but at 14 wk there are trends for FPR2 KO (*p* = 0.062) and ChemR23 KO (*p* = 0.085) animals to freeze less than WT.

There were no differences among genotypes in CON animals, but in ALC animals there was a trend for FPR2 KO to freeze more than WT or ChemR23 KO (*p* = 0.080 and *p* = 0.094, respectively). Within genotype, only ChemR23 KO animals showed an effect of exposure with less freezing in ALC compared with CON (*p* = 0.004).

There was a positive correlation between percent time freezing in context and percent time freezing to the cue across all animals (*r* = 0.384, *p* < 0.001). Within genotypes, this was seen in WT (*r* = 0.417, *p* < 0.001) and ChemR23 KO (*r* = 0.509, *p* < 0.001), but not in FPR2 KO mice.

#### 3.5.6. Correlations

Correlations between body weight and behavior: For the whole dataset, body weight positively correlated with percent time in closed arms of EPM (*r* = 0.346, *p* < 0.001) and percent time freezing to the cue (*r* = 0.30, *p* < 0.001) and negatively correlated with distance traveled in EPM (*r* = −0.181, *p* = 0.003), grip strength (*r* = −0.189, *p* = 0.002), and activity in NOR (*r* = −0.44, *p* < 0.001). For three of these, the correlations were significant or trends in all genotypes: the positive correlations with percent time in closed arms of EPM and percent time freezing to the cue, and the negative correlation with activity in NOR (see Table 4 for *r*- and *p-*values).

**Table 4:** Pearson correlation outcomes.

Correlations with body weight	All mice	WT	FPR2 KO	ChemR23 KO
% time in closed arms of EPM	*r* = 0.266, *p* < 0.001	*r* = 0.190, *p* = 0.08^T^	*r* = 0.294, *p* = 0.008	*r* = 0.295, *p* = 0.003
% time freezing to cue	*r* = 0.310, *p* < 0.001	*r* = 0.510, *p* < 0.001	*r* = 0.186, *p* = 0.099 ^T^	*r* = 0.189, *p* = 0.059 ^T^
Correlations with grip strength				
% time in closed arms of EPM	*r* = −0.224, *p* < 0.001		*r* = −0.387, *p* < 0.001	
Distance in EPM	*r* = 0.205, *p* < 0.001	*r* = −0.223, *p* = 0.037	*r* = 0.392, *p* < 0.001	
Time on rotarod			*r* = −0.221, *p* = 0.049	
Activity in NOR	*r* = 0.240, *p* < 0.001	*r* = 0.227, *p* = 0.033	*r* = 0.324, *p* = 0.003	*r* = 0.203, *p* = 0.043
% time freezing to cue			*r* = −0.268, *p* = 0.016	

EPM, elevated plus maze; FPR2, formyl peptide receptor 2; KO, knockout; NOR, novel object recognition test; WT, wild type. T denotes trend, 0.05 < *p* < 0.1.

Grip strength is a non-invasive test of muscle strength (e.g., Takeshita et al., 2017), therefore we used Pearson correlation analysis to assess whether genotype differences in muscle strength were associated with other behavioral outcomes. When all animals were assessed together, grip strength positively associated with distance traveled in the EPM (*r* = 0.147, *p* = 0.016) and activity in NOR (*r* = 0.24, *p* < 0.001) and negatively correlated with percent time in closed arms of EPM (*r* = −0.276, *p* < 0.001). Only the positive association with activity in NOR was apparent in all genotypes (see Table 4 for *r*- and *p*-values).

Because the poor performance of FPR2 KO mice on grip strength may reflect an effect on skeletal muscle, we tested associations between grip strength and other behaviors for this genotype. When both ages were assessed together, grip strength positively correlated with movement in the EPM and activity in NOR, and negatively associated with percent time in closed arms of EPM, time on rotarod, and freezing in response to the cue (see Table 4 for *r*- and *p-*values). When each age was examined separately, grip strength positively correlated with movement in the EPM (*r* = 0.431, *p* = 0.005) and showed a trend to positively correlate with activity in NOR (*r* = 0.302, *p* = 0.058) at 6 wk but not at 14 wk. Similarly, the negative correlation between grip strength and freezing in response to the cue was apparent at 6 wk (*r* = −0.343, *p* = 0.031) but not 14 wk.

## 4. Discussion

To date, studies of FPR2 and ChemR23 and their ligands have emphasized their resolution activities in diverse acute and chronic inflammatory diseases and little attention has been given to their potential influences on cognition and behavioral outcomes despite their presence in the brain. We report here that both FPR2 and ChemR23 are essential for healthy brain development, and their loss-of-function leads to greater measures of anxiety and perhaps deficits in memory. Moreover, this is the first demonstration that they interact with alcohol to further alter behavioral performance, thus supporting their importance in attenuating the behavioral deficits associated with PAE. These alterations differ between the two knock-out lines, further highlighting that these receptors make distinct mechanistic contributions to these processes. Taken together, these findings emphasize the importance of FPR2 and ChemR23 in the brain and offer mechanistic insight into how omega-3 PUFAs support healthy brain development and may act to improve the behavioral defects that characterize PAE.

With respect to behavior, adolescent female ChemR23 KO mice froze more than WT animals in a fear conditioning test. Although the WT animals showed more freezing in the fear conditioning test as young adults compared with adolescence, this age- or experience-dependent improvement was not observed in ChemR23 KOs and may suggest problems with long-term memory in those animals. The adolescent FPR2 KO animals froze more than WT in the fear conditioning test and did not show increased freezing between adolescence and young adulthood. ChemR23 KO females showed less anxiety-like behavior in the elevated plus maze in young adulthood; they spent less time in closed arms than other mice but did not show any difference in the number of entries into open arms or greater activity in the maze. Unlike a previous report (Gallo et al., 2014), there was no evidence of differences in anxiety-like behavior or changes in NOR in FPR2 KO males compared with WT mice. Moreover, the young adult FPR2 KOs performed poorly on a test of grip strength which suggested an effect on skeletal muscle, and they had the lowest activity in both EPM and NOR, however, they performed normally on the rotarod and were not different to the ChemR23 KO mice in fear conditioning. Correlation analyses were performed to try to understand whether the potential skeletal muscle effect altered other behaviors in the FPR2 KO mice; correlations between grip strength and other behaviors were age-dependent and were only seen in adolescent mice even though grip strength worsened with age suggesting that at least some of the behavior deficits were independent of grip strength.

Interestingly, FPR1 loss-of-function is associated with reduced anxiety and fear behaviors (Gao et al., 2011), suggesting that FPR1 and FPR2 receptors serve complementary roles. Although the mice tested here are global knock-outs, insight into the affected population emerges from the well-mapped neurocircuitry of fear-conditioning, which involves the amygdala, medial prefrontal cortex, and hippocampus (Shin and Liberzon, 2010), regions where microglia express both FPR2 and ChemR23. Microglia are known to affect neurobehaviors and their function can be influenced by diet (see review by Paolicelli and Ferretti, 2017); for example, deficiency of omega-3 PUFAs during development alters microglial motility and expression of phenotypic markers and increases expression of pro-inflammatory cytokines in hippocampus at P21 (Madore et al., 2014).

Alcohol, including in the context of prenatal exposure, is strongly pro-inflammatory, and this inflammation is a significant driver of alcohol-related pathologies including the behavioral deficits that typify FASD (Cantacorps et al., 2017; Komada et al., 2017; Bodnar et al., 2020; Gano et al., 2020). Both FPR2 and ChemR23 are implicated in inflammatory brain disorders including ischemic stroke and Alzheimer’s (Valente et al., 2022), and findings herein extend those actions to PAE. Loss of either receptor further altered PAE’s impact in select domains involving anxiety, learning, and activity. That alcohol’s impact was a function of genotype suggests the involvement of different cell types and/or different functions. The relatively modest changes reported here reflects our use of a relatively moderate exposure to avoid basement effects in the alcohol × genotype interaction; genotype did not affect alcohol levels *per se*.

Where and how FPR2 and ChemR23 act to affect behavior, and how they interact with PAE, is unclear. Using whole transcriptome sequencing, we find that FPR2 is present in fetal liver but not fetal brain or placenta (Petry et al., unpublished), suggesting it may affect behavior indirectly through peripheral inflammation. Alcohol increases gut permeability and the entry of pro-inflammatory bacterial products including LPS and cytolysin (Duan et al., 2019); in our model alcohol increases the content of microbial products in both dam and fetus (Virdee et al., 2021). A loss of FPR2 would heighten the inflammatory responses to these products, thereby exacerbating alcohol’s damage and limiting the capacity for recovery (Dufton and Perretti, 2010; Sansbury and Spite, 2016). In contrast, we detected ChemR23 in the E17.5 WT mouse brain and liver, and in the placenta, and alcohol induced its brain expression 1.17-fold (Padj = 0.032). ChemR23 loss-of-function leads to a similar pro-inflammatory environment (Cash et al., 2008; Zhang et al., 2018) that is accompanied by cognitive impairment (Zhang et al., 2022). In the brain it acts to recruit and activate microglia and promotes their transition to a pro-resolving phenotype in models of brain injury (Zhang et al., 2018). Thus, ChemR23 may serve to limit alcohol’s pro-inflammatory actions on microglia.

Limitations of this study include the global nature of these knockouts, which precludes identification of the cell population mediating these behavioral deficits. Although all mice used the same background strain (C57BL/6J), each line was generated independently from WT-WT or KO-KO breedings. As such, it is possible that environmental differences, including maternal care or litter effects, could contribute to genotype-related differences in growth or behavior. Off-target CRISPR effects also would not be detected. Similarly, it is unclear whether these receptors are acting within the fetus, maternal compartment, or both at the time of alcohol exposure. Also unknown is the identity of the ligands that stimulate these receptors’ protective actions in response to alcohol; however, the candidate lipoxins, cytokines, and formylated microbial peptides are consistent with alcohol’s known actions, and it is possible that multiple signals act in concert. Future studies could also examine other ligands for these receptors, such as chemerin and annexins. It should also be noted that animals underwent all behavioral testing at both ages which can add a test–retest confound, however, tests were administered with at least 5 weeks between them and studies in rodents find that a 4-week intertrial interval restores the baseline behavior for EPM and that NOR is repeatable regardless of the length of the intertrial interval (Cnops et al., 2022).

In summary, we validate and expand the range of behavioral domains influenced by FPR2 and ChemR23, consistent both with the role of inflammation in affecting behavioral performance and offering insight into the mechanism by which essential fatty acids support brain development. Findings also inform how supplemental PUFAs attenuate the cognitive impairments caused by PAE, and suggest that strategies that activate these receptors, such as RvD and RvE resolvins, could limit or improve recovery from alcohol’s damage.

## Data availability statement

The raw data supporting the conclusions of this article will be made available by the authors, without undue reservation.

## Ethics statement

The animal study was reviewed and approved by the Institutional Animal Care and Use Committee, David H. Murdock Research Institute.

## Author contributions

SM and SMS designed the study and wrote the first draft of the manuscript. SM, SRS, and SMS contributed to the conception of the study. EB, MM, and CM performed the study. SM performed the statistical analysis. All authors contributed to manuscript revision, read, and approved the submitted version.

## Funding

This work was supported by the NRI internal grant funds to SM and SMS, and by NIH (R01AA024980) to SM, (R01AA011085) to SMS, (R01ES031378) to SRS. The UNC Animal Models Core Facility was supported in part by P30 CA016086 Cancer Center Core Support Grant to the UNC Lineberger Comprehensive Cancer Center.

## Conflict of interest

The authors declare that the research was conducted in the absence of any commercial or financial relationships that could be construed as a potential conflict of interest.

## Publisher’s note

All claims expressed in this article are solely those of the authors and do not necessarily represent those of their affiliated organizations, or those of the publisher, the editors and the reviewers. Any product that may be evaluated in this article, or claim that may be made by its manufacturer, is not guaranteed or endorsed by the publisher.

## Footnotes

^1^mouse.brain-map.org

^2^brainrnaseq.org
