# Supplementary material for: Behavioral changes in FPR2/ALX and Chemr23 receptor knockout mice are exacerbated by prenatal alcohol exposure
Source: Front Neurosci. 2023 Jul 6;17:1187220. doi: 10.3389/fnins.2023.1187220 (PMC10357512; doi:10.3389/fnins.2023.1187220)
Supplement: Supplementary file 1 [file Data_Sheet_1.docx]

**Supplemental Figure 1. PCR Genotyping of Wild-Type C57BL/6J (WT), FPR2 Knock-out, and ChemR23 Knock-out mice.**


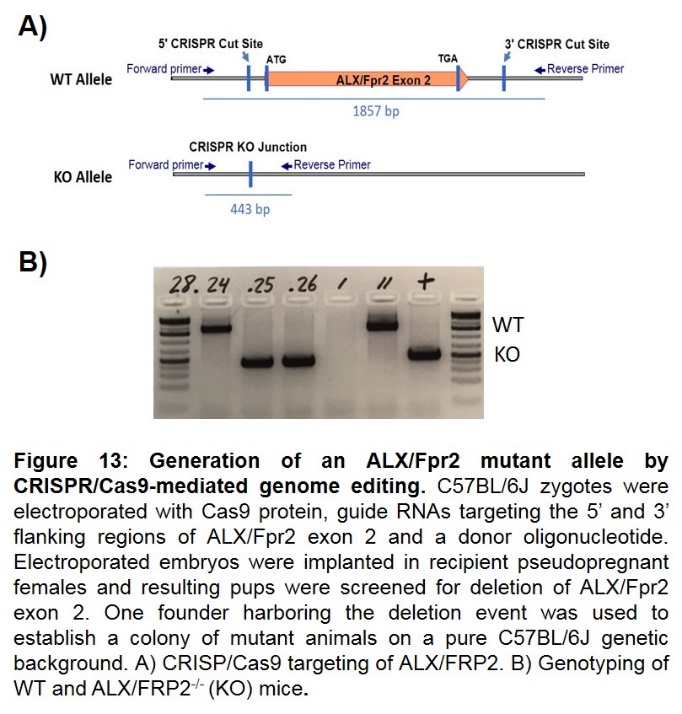


**
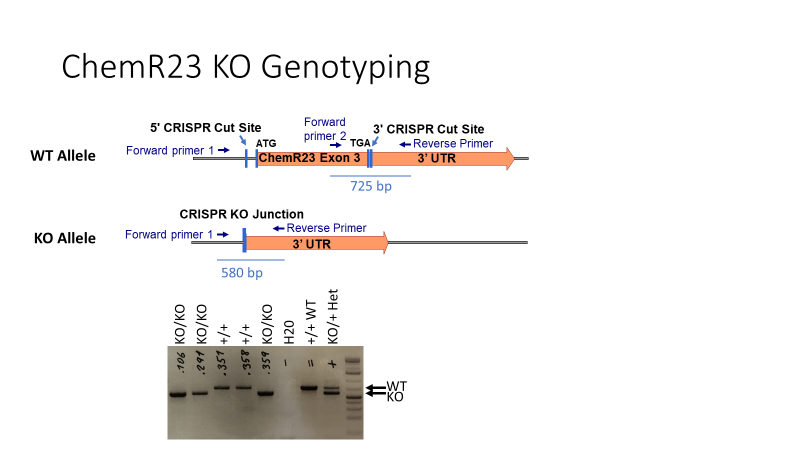
**

Supplemental Figure 1: TOP and MIDDLE: Schematics showing generation of the knock-out mice. (Reproduced with permission from D.O. Cowley, UNC Animal Models Core Facility.)

BOTTOM: PCR genotyping of FPR2 and ChemR23 knock-out mice, and the parent C57BL/6J parent (wild-type, WT) strain. The WT strain yields two PCR products, the full length ChemR23 (725 bp) and FPR2 (433 bp) fragments. The ChemR23 knock-outs have a truncated fragment of 580 bp, and the FPR2 knock-outs yield a 238 bp truncated fragment.

The PCR genotyping primers are:

FPR2: Fpr2-3ScF1 5'-TTCTGCCTTCCTTACCTTATGC-3' forward

Fpr2-Del-F1 5'-CTGTGAAAATGCTCTCCTGTATCA-3' forward

Fpr2-3ScR1 5'-GCAAATGCGTATGAGTATAAATGC-3' reverse

ChemR23: Cmklr1-5ScF1 5'-GGAGCAGGAAACAGAATAGGAC-3' forward

Cmklr1-3ScF1 5'-ATCACCTTCTTCCTCTGCTGG-3' forward

Cmklr1-3ScR1 5'-GGTTTGACTGTCATGTTGCCATA-3' reverse

**Supplemental Figure 2. Partial gene sequence for FPR2 Knock-out mice**

AGAAATGTGGAGAGCTATGCTACCCCAGAAAGGTATGCAGTAAAGTGCTAGTTTTGAAAT

GTTACTGTGAAAATGCTCTCCTGTAGTAGCAGGTGGTATACATTCTAAATGAGTGTCATG

TCAGAAGGAGCCAAATATCTGAGAAATGGTTGTTTTTGAAAACTTTCAG**GTGCAGACAAA**

**ATGGAATCCAACTACTCCATCCATCTGAATGGATCAGAAGTGGTGGTTTATGATTCTACC**

**ATCTCCAGAGTTCTGTGGATCCTCTCAATGGTGGTTGTCTCCATCACTTTCTTCCTTGGT**

**GTGCTGGGCAATGGACTAGTGATTTGGGTAGCTGGATTCCGGATGCCACACACTGTCACC**

**ACTATCTGGTATCTGAATCTAGCATTGGCTGACTTTTCTTTCACAGCAACTCTACCATTC**

**CTTCTTGTTGAAATGGCTATGAAAGAAAAATGGCCTTTTGGCTGGTTCCTGTGTAAATTA**

**GTTCACATTGTGGTAGATGTAAACCTGTTTGGAAGTGTCTTCTTGATTGCTCTCATTGCC**

**TTGGACCGCTGCATTTGTGTTCTGCATCCAGTCTGGGCT****CAGAACCACCGCACTGTGAGC**

**CTGGCTAGGAAGGTGGTTGTTGGGCCCTGGATTTTTGCTCTGATTCTCACTTTGCCCATT**

**TTTATTTTCTTGACTACTGTTAGAATTCCTGGAGGAGATGTGTATTGTACATTCAACTTT**

**GGATCCTGGGCTCAAACTGATGAAGAAAAGTTGAACACAGCTATCACTTTTGTAACAACT**

**AGAGGGATCATCAGGTTCCTTATTGGTTTCAGCATGCCCATGTCAATTGTTGCTGTTTGC**

**TATGGACTCATTGCTGTCAAGATCAACAGAAGAAACCTTGTTAATTCCAGCCGTCCTTTA**

**CGAGTCCTTACAGCAGTTGTGGCTTCCTTCTTTATCTGCTGGTTTCCCTTTCAGCTTGTG**

**GCCCTTTTGGGCACAGTCTGGTTTAAAGAGACATTGCTTAGTGGTAGTTATAAAATTCTT**

**GACATGTTTGTTAACCCAACAAGCTCATTGGCTTACTTCAATAGTTGTCTCAATCCGATG**

**CTCTATGTTTTCATGGGCCAGGACTTTCGTGAGAGATTTATTCATTCCCTGCCTTATAGT**

**CTTGAGAGAGCCCTGAGTGAGGATTCTGGTCAAACCAGTGATTCAAGCACCAGTTCTACT**

**TCACCTCCTGCAGACATTGAGTTAAAGGCCCCATGAAGTCTGTGATAAGGGATGGTTTTC**

**ATTCCACTTCAGTCCCATTCCACTTCTGCCTTCCTTACCTTATGCTGTGTCCTATAGCAT**

**TTTTAATCTGAAAAAAAAAAAAAAACTTCTGTGTCCCCTGAATCTGGAAAATGAAATAAA**

**AGTCAATGT**TAATATTGTTGTTCTTTTGTGTTCTTTTAAAAACTAAGCCTTTATACTCTA

AACAAAACTAATGTAGTGGAGCAATGTACTACAAGAGAGGAAAAGAGCAATGCATATTCT

CAAGGCTTATACATGATGGTAAATATTAAAAATAAGTTATTCTATTTCATAGGCTGAGAA

GATGTCTCAATGGTTAATACAAATTTCTGCTTTAGTAGAATACCTGAGTTTAGTTTCAAC

CACCCACAGCACCTGAATTACAATTGGTAGTAATTCTATCTGTAGAAAATTCAAGGGCTC

Supplemental Figure 2: In the partial gene sequence from EnsEMBL for FPR2 (ENSMUST00000064068.5), exon 2 is identified by bold font and orange highlights. The guide RNAs used in the genome editing for FPR2 KO mice were 5sg74B (protospacer sequence 5’- gATACCACCTGCTACTAC-3’) highlighted in green and 3sg47T (protospacer sequence 5’-GAGCAATGCATATTCTC-3’) highlighted in blue.

**Supplemental Figure 3. Partial gene sequence for ChemR23 Knock-out mice**

AACAGGAGGAGGAGCAGGAAACAGAATAGGACTGAGAAGATGGATAGATGAACTAATTAA

CCTTCATGTTCAGTGAGAGACCCAGTCTTCTCTGACATTCCCCTCTGCCCTCCACACTTG

CCTACATGAGCATATGTACACGCACATACACACACAGGTGTACATGCACATATACAAACA

GGCATACGAATGCAAAATAAAGACAAGAAATGGCAAAAGGGGAGATCGTTCACAACCCAG

GCCTTGCTCAGATGAGGAGGTGCCAGGTGGTGTCTCAGCACCTGTCCACAGAGGTCCTCA

GCCTGTGACCCTGTCTTCCCTCACAG**AGATGGAGTACGACGCTTACAACGACTCCGGCAT**

**CTATGATGATGAGTACTCTGATGGCTTTGGCTACTTTGTGGACTTGGAGGAGGCGAGTCC**

**GTGGGAGGCCAAGGTGGCCCCGGTCTTCCTGGTGGTGATCTACAGCTTGGTGTGCTTCCT**

**CGGTCTCCTAGGCAACGGCCTGGTGATTGTCATCGCCACCTTCAAGATGAAGAAGACCGT**

**GAACACTGTGTGGTTTGTCAACCTGGCTGTGGCCGACTTCCTGTTCAACATCTTTTTGCC**

**GATGCACATCACCTACGCGGCCATGGACTACCACTGGGTGTTCGGGAAGGCCATGTGCAA**

**GATCAGCAACTTCTTGCTCAGCCACAACATGTACACCAGCGTCTTCCTGCTGACTGTCAT**

**CAGCTTTGACCGCTGCATCTCCGTGCTGCTCCCCGTCTGGTCCCAGAACCACCGCAGCAT**

**CCGCCTGGCCTACATGACCTGCTCGGCCGTCTGGGTCCTGGCTTTCTTCTTGAGCTCCCC**

**GTCCCTTGTCTTCCGGGACACCGCCAACATTCATGGGAAGATAACCTGCTTCAACAACTT**

**CAGCTTGGCCGCGCCTGAGTCCTCCCCACATCCCGCCCACTCGCAAGTAGTTTCCACAGG**

**GTACAGCAGACACGTGGCGGTCACTGTCACCCGCTTCCTTTGCGGCTTCCTGATCCCCGT**

**CTTCATCATCACGGCCTGCTACCTTACCATCGTCTTCAAGCTGCAGCGCAACCGCCTGGC**

**CAAGAACAAGAAGCCCTTCAAGATCATTATCACCATCATCATCACCTTCTTCCTCTGCTG**

**GTGCCCCTACCACACCCTCTACCTGCTGGAGCTCCACCACACAGCTGTGCCAAGCTCTGT**

**CTTCAGCCTGGGGCTACCCCTGGCCACGGCCGTCGCCATCGCCAACAGCTGCATGAACCC**

**CATTCTGTACGTCTTCATGGGCCACGACTTCAGAAAATTCAAGGTGGCCCTCTTCTCCCG**

**CCTGGCCAACGCCCTGAGTGAGGACACAGGCCCCTCCTCCTACCCCAGTCACAGGAGCTT**

**CACCAAGATGTCGTCTTTGAATGAGAAGGCTTCGGTGAATGAGAAGGAGACCAGTACCCT**

**CTGAACCTCACCTGGGAATGTCCCCCAAAGGTGCCACGGCCCAGGGACGCCTAGGGACTT**

**GTCTCCGGAAGTGGGAGACATGCCGGGAGCCTTTGGGAATGCTCCAATGCCCACTGAATT**

**TTGCACAAGGCGGCTCATGTTTTAAGTGGGGTTCCCAAGTGTGGACACTCTTCCAGTAAA**

**ATGGCAGGCAAGCAACCCGAGCCTCTACAACAGGAGCAGGGGACCGACTGTGACTGACTC**

**AGAAAAGGGAGCATCTCTGAAGCCAAGACTTGAGCTGTGACCAACATACAGGCCAACATA**

**CACGATGTCGCCGTGCATGCCCTGAACATGCTGCGCAGTCTTCGTGGGTGAGGAAGTTAC**

**CGCAAACCCATTGCAGACCTGTTATGGCAACATGACAGTCAAACCAACAAAGCCCACTAC**

**ACCCCAACATCCTCCAAGACCTTGACTTTGGATTTCAGAAGAACGGGGGGTGGGGGGAAC**

**GAGGACCTGAGGGGCTTCATGGAACTTTGCAAGGGCAACACAGGGTTCTGTGTGTGTGTG**

**TGTGTGTGTGTGTGTGTGTGTGTGTGTGTGTGTGTGGTAAAGAGGAGGAGACGGAGAGCA**

**GAGAATCCCAAAGGATGTGGGTACAGGATATCTTACACCATCATGCCACGGAGGAGACAG**

**AGAGCTATAGTGGTGGTTGCTTGGGGGTTCAGGTGGTGGCAGCTCTTTGCTTCAGTGTGT**

**ATACTCACTTAGCACTGAACCCTGAGGCTGGGTGGAGGCCTTTTCACAGAAGACTCCATC**

**TGATCCCATCCCATCCCCTCCCCTCCCCTCCCCTCCCCTCCCCTCCCCTCCCCTCCCCTC**

**CCCTCCCCTCCCCTCCCAGGGTCTCTTCTCCCCTAATCCCCTCAATGCTGGCTGGAAAAA**

**GGGCTTAGAATGTCCCAGAGACTGGTCTAGAACGGAGTTTTGGGACTCCATCTTCAGAGT**

**GTCTTCCCAAGGCTGTCTGGCTGGTCATCTGGGCAGCTGACCAGCTTTCCCGAGTCCTCC**

**TTGAATGTATCAACAGCCCCGTGGGCAGCCCAGGATCAGGCTTTCCTCCCCTGGCAGGAA**

**AATGGCTCACTCACCCCCCCCCCACACACACACACTGTTCATCAGTAGCAAAAGACCCGA**

**GCCCTGCTCCGTCTGGCTTGACATCATTCCTCATGGAGGAGGGGTGGTGTGCACTTCGTT**

**AGCATGTCCCATGCATGTTAAGTATTTCACATGCACCTTCTCATTTATTCTTCACAGAAA**

**CCTCATATGTATGAGGACAAGCAGAGACTCAGAGAGGTTAAGCAACTGGCCCGAGGGGAC**

**CCAGTTAGATGTTGGTAGAGACTGGATTCGAATCGCAGTGGGCTTGGCTCGAAGGAAGAT**

**GAAGAGTCTGGGGAAACTAATAGGTTGGGGTTTTAGCCATAAATGCTGTTTTGTTCTGTT**

**TTTTCCATGTAAACATCTTAATGGTCTTTTTGA**CGTCTTGCTTGTTGTTTTCAGAGTAAT

ACAATATGCAAAGAAAAGGCGTACTCTCCAGCCCCAGCCTCCCTGGCTCCCATCACACAC

ACCCTGAAATCTGCTAGGCAAGCAGAGGGAGGGAGCCTGGTAGGTCAGCCTGTCCAGTCC

AAGGTCTTCTGGCCTGCAGTGTCCTCTGTTCCCTTCCAAGCCACTTTTCCACCGGGGTGT

AACACACAGATGTGTGTCCTCCGGCCCCAGCCAGTTCCCGTATTATAGACTGCTGGGAGT

CACACTTCATCAGGGGGACAGATTGTTCCTCCCTGGTCGGGTCTTAAGATGAGGCAGTTC

TGAACAGATCCAAGCTGGAACCTCTGGCTTCTGAGAGAGCCCAAACAACCAAGGATTGGG

AGCAGTAAGGGTGGTTTAAAAATAGCCTTGTGTTCCCATTGTCACCCTCTCCGCGACTCC

CCAGGGCTACAGATGTGCAACTGAGGCGTGCCACGGTATGGGATTCATCATGTCAACGGA

GGCGCCTGTCCGTGACTGGGGCCACATGGTGTCTTGGACAGTGCACTTCCCGGACATTGG

GGCTGACCAGGTCAGGCAGGGGACCAGGAAATC

Supplemental Figure 3: In the partial gene sequence from EnsEMBL for CMKLR1 (ChemR23; ENSMUST00000047936.13), exon 3 is identified by bold font and orange highlights. The guide RNAs used in the genome editing for ChemR23 KO mice were 5sg81T (protospacer sequence 5′‐ GAGATCGTTCACAACCC‐3′) highlighted in green and 3sg81T (protospacer sequence 5′‐gCGGCCCAGGGACGCCTA‐3′) highlighted in blue.
